# Why does feedback work? Unpacking the serial mediation of emotion regulation and discourse skills in mathematics

**DOI:** 10.3389/fpsyg.2026.1854668

**Published:** 2026-06-10

**Authors:** Mohong Wu, Yekun Liu, Juncheng Guo, Othman Talib

**Affiliations:** 1 Union Committee, Cangzhou Normal University, Cangzhou, Hebei, China; 2 School of Physics and Information Engineering, Cangzhou Normal University, Cangzhou, Hebei, China; 3 School of Education, Jingchu University of Technology, Jingmen, Hubei, China; 4 Faculty of Social Sciences and Liberal Arts, UCSI University, Kuala Lumpur, Malaysia

**Keywords:** emotion and motivation self-regulation, feedback literacy behavior, longitudinal design, mathematical higher-order thinking, mathematics discourse feedback skills, serial mediation

## Abstract

**Introduction:**

Cultivating mathematical higher-order thinking is a paramount pedagogical objective. While student feedback literacy has gained attention, its longitudinal mechanisms on complex cognitive outcomes remain underexplored. This study investigates the sequential mediating roles of emotion and motivation self-regulation and mathematics discourse feedback skills in the relationship between feedback literacy behavior and mathematical higher-order thinking.

**Methods:**

A three-wave longitudinal design with a 3-month interval was employed. A total of 1,795 Chinese high school students completed surveys assessing feedback literacy behavior at Time 1, emotion and motivation self-regulation at Time 2, and mathematics discourse feedback skills alongside mathematical higher-order thinking at Time 3. Data were analyzed using structural equation modeling, controlling for autoregressive effects.

**Results:**

Feedback literacy behavior at Time 1 was significantly and positively associated with mathematical higher-order thinking at Time 3. Both emotion and motivation self-regulation and mathematics discourse feedback skills independently mediated this relationship. Furthermore, the serial mediation pathway was significant: feedback literacy behavior enhanced emotion and motivation self-regulation, which subsequently fostered mathematics discourse feedback skills, which were concurrently associated with mathematical higher-order thinking.

**Discussion:**

Feedback literacy behavior acts as a developmental catalyst for cognitive restructuring. This trajectory is sequentially mediated by intra-individual psychological regulation and interpersonal social interaction. Educators should transcend traditional corrective feedback by actively cultivating feedback literacy and facilitating dialogic feedback environments.

## 1 Introduction

In contemporary mathematics education, cultivating students' mathematical higher-order thinking has emerged as a paramount pedagogical objective (Ismail et al., 2024). As students transition into senior high school, the curriculum demands a fundamental shift from routine procedural calculations to complex problem-solving, critical analysis, and creative reasoning (Dorner et al., 2025). Mathematical higher-order thinking is not only essential for achieving academic excellence but also a critical skill required for navigating an increasingly complex and information-rich society (Santos et al., 2025). However, many students struggle with this cognitive transition, highlighting an urgent need for effective instructional strategies and self-regulatory mechanisms to facilitate deep learning (Xue et al., 2025).

Formative feedback has long been recognized as a powerful catalyst for student learning and cognitive development (Brown et al., 2025). Yet, traditional feedback paradigms often position students as passive recipients of teacher-generated information, a transmission model that frequently fails to translate into meaningful cognitive gains (Nieminen et al., 2026). To address this shortcoming, contemporary educational theory has pivoted toward student feedback literacy, highlighting the learner's active agency in soliciting, interpreting, and applying feedback (Carless and Winstone, 2023). While extensive research has established the positive impact of feedback literacy on general academic achievement, its specific, long-term influence on the development of complex cognitive outcomes, such as mathematical higher-order thinking, remains underexplored (Gong et al., 2025).

Furthermore, the underlying mechanisms through which feedback literacy behavior fosters higher-order thinking require deeper investigation, particularly from a longitudinal perspective (Zhang et al., 2023). The development of advanced mathematical reasoning is not an instantaneous event but a cumulative, dynamic process (Menon and Chang, 2021). Existing literature often examines the affective, behavioral, and cognitive dimensions of learning in isolation (Estévez et al., 2021). However, integrating frameworks such as self-regulated learning and control-value theory suggests that active behavioral engagement with feedback likely triggers a cascade of psychological and social processes (Hwang, 2025; Safferthal and Prinz-Weiß, 2025). Specifically, it remains unclear how students' proactive feedback behavior interacts with their internal emotion and motivation self-regulation, and how this psychological stability subsequently empowers their participation in social learning contexts, such as engaging in constructive mathematics discourse.

To address these theoretical and empirical gaps, the present study proposes a comprehensive longitudinal model to unravel the developmental trajectory from feedback literacy behavior to mathematical higher-order thinking. Focusing on Chinese high school students, a population operating within a highly competitive educational context where mathematics achievement is heavily emphasized and often accompanied by significant academic pressure, this study investigates the sequential mediating roles of intra-individual psychological regulation and interpersonal social interaction (Sun et al., 2025). Utilizing a three-time-point longitudinal approach, the present study seeks to test a sequential mediation framework. This method offers deeper insights into the mechanisms through which active feedback utilization establishes the cognitive foundations essential for complex mathematical problem-solving.

### 1.1 Feedback literacy behavior and mathematical higher-order thinking

Feedback literacy behavior refers to the proactive actions students take to make sense of, act upon, and elicit feedback information rather than merely receiving it passively (Carless and Boud, 2018). In the context of high school mathematics education, the curriculum shifts toward increasingly abstract concepts and complex problem-solving (Niss and Højgaard, 2019). Consequently, the transition from routine procedural calculation to higher-order thinking, which is characterized by analysis, evaluation, and creation, requires students to constantly refine their existing cognitive schemas (Aydin and Birgili, 2023).

Self-regulated learning (SRL) theory provides a robust theoretical lens for understanding the link between feedback literacy behavior and cognitive development (Zimmerman, 2002). According to SRL, feedback serves as a critical regulatory mechanism that signals the discrepancy between current performance and the intended learning goal (Hattie and Timperley, 2007). However, the mere presence of feedback is insufficient; it is the student's behavioral engagement with feedback (i.e., feedback literacy behavior) that facilitates cognitive restructuring (Winstone et al., 2017). When students actively engage in feedback processing behaviors, such as identifying errors, seeking clarification, and revising strategies, they are effectively engaging in “deep processing” (Cheng and Liu, 2022).

Specifically, mathematical higher-order thinking involves cognitive tasks that cannot be resolved through rote memorization or mechanical drills (Gravemeijer et al., 2017). Feedback literacy behavior prompts students to reflect on why a mathematical solution failed rather than just correcting the answer (Loibl and Leuders, 2019). This iterative process of reflection and correction fosters a deeper conceptual understanding and metacognitive awareness, which are antecedents to higher-order thinking skills (Yan, 2020). Therefore, students who exhibit higher levels of feedback literacy behavior at an earlier stage are more likely to develop the cognitive scaffolds necessary for subsequent improvements in mathematical reasoning. Based on the theoretical arguments above and the longitudinal nature of learning accumulation, we propose the following hypothesis:

**H1** Feedback literacy behavior at Time 1 is positively associated with mathematical higher-order thinking at Time 2 and Time 3.

### 1.2 The mediating role of emotion and motivation self-regulation

In the high-pressure environment of Chinese high school education, where mathematics achievement is often linked to significant academic consequences, the ability to regulate emotions and motivation is pivotal (Zhang and Wang, 2020). Emotion and motivation self-regulation refers to the strategies students use to initiate, maintain, or adjust their affective states and motivational beliefs to achieve learning goals (Stockinger et al., 2025).

Control-value theory (Pekrun, 2024) suggests that achievement emotions are determined by students' cognitive appraisals of control and value. Feedback literacy behavior acts as a critical antecedent to these appraisals. When students proactively engage with feedback, such as analyzing errors or seeking teacher guidance, they shift from a passive state of confusion to an active state of problem-solving (Molloy et al., 2020). This behavioral engagement enhances their sense of agency and control over their learning process (Nieminen and Tuohilampi, 2020). Consequently, students who actively utilize feedback are better equipped to down-regulate negative emotions, such as anxiety regarding difficult mathematical concepts, and up-regulate autonomous motivation (Wisniewski et al., 2020).

Furthermore, the impact of these regulatory processes extends to cognitive outcomes. According to the resource allocation model (Field and Klingert, 2001), emotion regulation and cognitive processing compete for limited working memory resources. Mathematical higher-order thinking involves complex cognitive operations that require substantial attentional resources (Caviola et al., 2014). If students fail to regulate negative emotions or sustain motivation, their cognitive resources are consumed by irrelevant ruminations or anxiety (Barroso et al., 2021). Conversely, effective emotion and motivation self-regulation preserves these cognitive resources, which allows students to direct their attention toward deep processing, logical reasoning, and creative problem-solving (Camacho-Morles et al., 2021).

Therefore, feedback literacy behavior not only directly influences cognition but also operates indirectly by fostering a regulated psychological state that is conducive to complex learning. Consequently, we formulate the following prediction:

**H2** Emotion and motivation self-regulation at Time 2 mediates the relationship between feedback literacy behavior at Time 1 and mathematical higher-order thinking at Time 3.

### 1.3 The mediating role of mathematics discourse feedback skills

Mathematics discourse feedback skills denote the student's competence in engaging in constructive dialogues about learning evidence, specifically involving articulating mathematical reasoning, negotiating meaning with peers, and asking clarifying questions (Kontorovich, 2021). Beyond the internal emotional pathway, feedback literacy behavior also facilitates cognition through a social-interactive mechanism (Nieminen and Carless, 2023).

First, feedback literacy behavior serves as a developmental precursor to mathematics discourse feedback skills. Feedback literacy is not merely a solitary act of correction but often involves social interactions, such as soliciting explanations from teachers or discussing errors with peers (Molloy et al., 2020). From a Sociocultural Perspective, learning is a process of participating in a community of practice (Forman and Mccormick, 1995). When Chinese high school students persistently engage in feedback literacy behaviors, such as actively asking clarifying questions or justifying their solution paths, they are effectively practicing the “language of mathematics” (Esterhazy, 2018). Over time, this sustained behavioral engagement cultivates refined discourse skills, which transforms tentative inquiries into proficient academic argumentation (Latifi and Noroozi, 2021).

Second, these acquired discourse skills are instrumental in fostering mathematical higher-order thinking. Drawing on Vygotsky's Social Constructivism, cognitive development proceeds from the inter-psychological plane (social interaction) to the intra-psychological plane, or individual cognition (Adams, 2006). Mathematics discourse feedback skills allow students to externalize their thinking processes (Tullis and Goldstone, 2020). By articulating their reasoning and responding to counter-arguments during feedback episodes, students are compelled to reorganize their cognitive schemas and detect logical gaps (Tullis and Goldstone, 2020). This process of “exploratory talk” directly supports the analysis, evaluation, and creation dimensions of higher-order thinking (Zhan et al., 2023).

Therefore, mathematics discourse feedback skills act as a bridge that translates the behavioral habit of engaging with feedback into the cognitive capacity for complex reasoning. Based on this reasoning, we propose the following hypothesis:

**H3** Mathematics discourse feedback skills at Time 3 mediate the relationship between feedback literacy behavior at Time 1 and mathematical higher-order thinking at Time 3.

### 1.4 The sequential mediation pathway

Finally, we propose that the influence of feedback literacy behavior on mathematical higher-order thinking operates through a sequential chain that integrates psychological regulation and social interaction. While a parallel mediation approach assumes that emotional regulation and discourse skills operate independently to facilitate cognition, such a model fails to capture the inherent developmental dependency between affective states and complex social behaviors. Sequential mediation is theoretically preferable for this study because it models learning as a progressive transformation of resources, where intra-psychological stability acts as a fundamental prerequisite for inter-psychological engagement. Specifically, the theoretical rationale for chronologically positioning emotion and motivation self-regulation prior to mathematics discourse feedback skills is rooted in the resource-intensive and socially risky nature of classroom discourse (Carless and Boud, 2018). The critical link in this sequential model lies in how internal regulation unlocks external participation.

According to Fredrickson's (2004) Broaden-and-Build framework, experiencing positive affect and well-regulated motivation expands a person's immediate cognitive and behavioral capacities, which subsequently fosters the development of lasting assets, such as interpersonal competencies. In the context of Chinese high school education, students often experience “foreign language anxiety” or the fear of “losing face” when discussing errors publicly (Jiang and Dewaele, 2019). Students with low self-regulation capabilities are prone to withdrawal behaviors to protect their self-esteem (Izadpanah and Charmi, 2022; Guo et al., 2025a). Conversely, feedback literacy behavior first establishes a sense of control, which enables effective emotion regulation (Nieminen et al., 2022). This regulated psychological state reduces social anxiety and safeguards the motivational resources required to engage in public discourse (Guo et al., 2025b).

Once students are psychologically emboldened to participate in mathematics discourse, they can utilize these skills to deepen their cognitive processing (Ing et al., 2015). As articulated in the discussion of H3, the act of verbalizing mathematical logic serves as the proximal mechanism for cognitive restructuring. Therefore, the complete causal chain suggests that feedback literacy behavior initiates a virtuous cycle: it fosters a regulated mindset that empowers social participation, which subsequently facilitates the deep cognitive processing required for higher-order thinking. Based on this integrated theoretical framework, we propose the final serial mediation hypothesis:

**H4** Feedback literacy behavior at Time 1 affects mathematical higher-order thinking at Time 3 through the serial mediation of emotion and motivation self-regulation at Time 2 and mathematics discourse feedback skills at Time 3.

### 1.5 The present study

Building upon the theoretical frameworks discussed above, the present study proposes a comprehensive longitudinal model to unravel the developmental trajectory from feedback literacy behavior to mathematical higher-order thinking. Specifically, this research focuses on Chinese high school students and employs a three-wave longitudinal design to empirically validate a serial mediation model. To rigorously isolate the directional relationships over time, autoregressive effects, including prior levels of mathematical higher-order thinking, are controlled for in the structural models.

**Figure 1:**
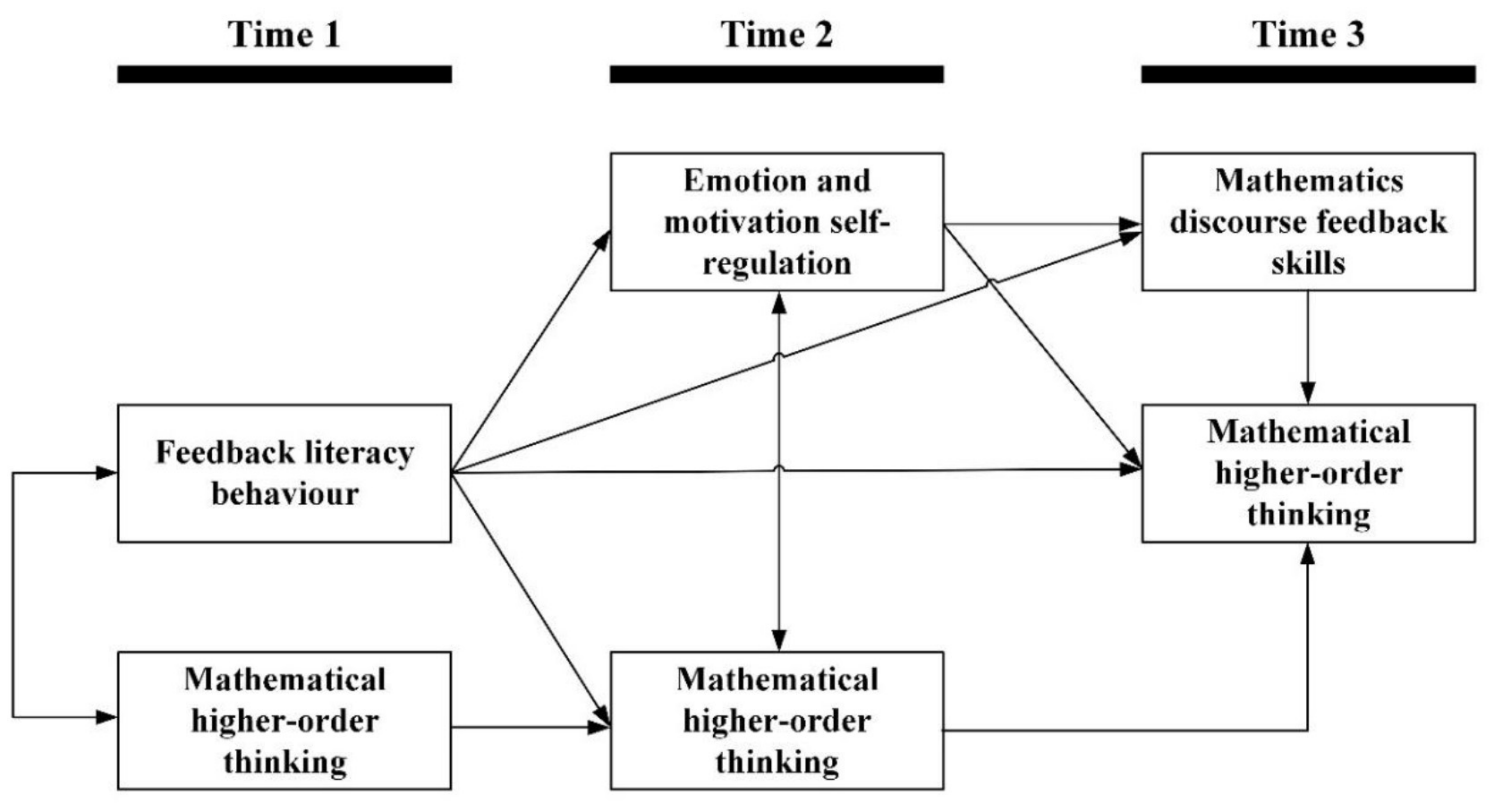
Hypothesized longitudinal serial mediation model linking feedback literacy behavior to mathematical higher-order thinking.

As illustrated in Figure 1, the proposed model integrates four primary hypotheses. First, H1 posits that feedback literacy behavior at T1 is positively associated with mathematical higher-order thinking at T2 and T3. Second, H2 proposes that emotion and motivation self-regulation at T2 mediates the relationship between feedback literacy behavior at T1 and mathematical higher-order thinking at T3. Third, H3 suggests that mathematics discourse feedback skills at T3 mediate the relationship between feedback literacy behavior at T1 and mathematical higher-order thinking at T3. Finally, representing the complete developmental chain, H4 posits that feedback literacy behavior at T1 affects mathematical higher-order thinking at T3 through the serial mediation of emotion and motivation self-regulation at T2 and mathematics discourse feedback skills at T3.

## 2 Method

### 2.1 Participants and procedure

During the baseline assessment (T1) in July 2025, an initial cohort of 1,795 adolescents (*M*_age_ = 16.88, SD = 1.14; age range: 15–19 years) completed the survey. Recruited through convenience sampling from three educational institutions in Hebei Province, China, the sample was fairly balanced by gender, comprising 918 males (51.1%) and 877 females (48.9%). The participants were distributed across three academic levels: Grade 10 (*n* = 740, 41.2%), Grade 11 (*n* = 820, 45.7%), and Grade 12 (*n* = 235, 13.1%). Demographically, urban residents accounted for 68.3% (*n* = 1,226) of the sample, while 31.7% (*n* = 569) lived in rural settings. Furthermore, an assessment of parental education revealed that over half held a bachelor's degree (53.2%), followed by those with an associate degree or less (41.3%), and a minority with postgraduate qualifications (5.5%).

Data collection spanned three distinct time points, separated by intervals of 3 months. The selection of this 3-month temporal spacing was both practically and theoretically driven. Practically, 3 months corresponds to exactly half an academic semester in the Chinese high school system (e.g., from the beginning of the term to mid-term examinations; Li et al., 2023). This timeframe encompasses a complete, dense pedagogical cycle characterized by continuous instruction, regular assignments, and comprehensive teacher feedback. Theoretically, acquiring mathematics discourse feedback skills and restructuring cognitive schemas for higher-order thinking are not instantaneous processes; they require sustained behavioral engagement and emotional adjustment (Liu et al., 2025). A 3-month interval provides sufficient time for the cumulative effects of feedback literacy behaviors to manifest in students' self-regulation and cognitive outcomes. Furthermore, this specific interval is optimal because shorter lags might capture transient state fluctuations rather than genuine developmental progression, whereas significantly longer intervals (e.g., one academic year) could introduce confounding maturation effects or structural changes in the learning environment (e.g., changing teachers or curricula; Dormann and Griffin, 2015). Data collection followed a standardized procedure across all waves.

At Time 2 (T2; October 2025), 1,436 students participated, representing a retention rate of 80.0% (attrition rate = 20.0%). At Time 3 (T3; January 2026), 1,220 students remained in the study. By the final assessment (T3), a 32.0% sample reduction was observed. This dropout was primarily driven by temporary unavailability on survey days (e.g., illness or competing extracurricular commitments) rather than institutional disenrollment, as all subjects maintained their school registration. To rule out the threat of attrition bias, we contrasted the baseline demographic profiles and initial variables of the retained cohort against those lost to follow-up. The absence of significant disparities between these groups supports the assumption that the data missingness mechanism is missing at random (MAR).

Prior to commencing the survey, ethical clearance was granted by the Institutional Review Board at the corresponding author's affiliated university. All research procedures strictly adhered to institutional guidelines and the core principles of the 1964 Declaration of Helsinki and its subsequent iterations. We successfully secured written informed consent from the legal guardians, alongside written assent directly from the adolescent participants. Furthermore, researchers explicitly briefed the students regarding the anonymous and voluntary nature of the investigation, emphasizing their unrestricted right to discontinue participation at any phase without facing negative consequences.

Questionnaires were administered in paper-and-pencil format within classrooms during regular school hours. Trained research assistants (e.g., school counselors familiar with the protocol) distributed the surveys and read standardized instructions to ensure consistency across all three waves. The administration process took approximately 20 min per wave. No financial rewards or course credits were provided for participation.

### 2.2 Measures

#### 2.2.1 Feedback literacy behavior (T1)

Feedback literacy behavior was assessed using the Chinese version of the Feedback Literacy Behavior Scale (FLBS), originally developed by Dawson et al. (2024) and validated for Chinese students by Zhu et al. (2025). This 24-item instrument captures five dimensions of students' enacted feedback literacy: Seek Feedback Information (five items; e.g., “I seek out examples of good work to improve my work”), Make Sense of Information (four items; e.g., “I carefully consider comments about my work before deciding if I will use them or not”), Use Feedback Information (five items; e.g., “When receiving comments, I plan how I will use them to improve my future work”), Provide Feedback Information (five items; e.g., “I offer to provide feedback to my peers”), and Manage Affect (five items; e.g., “I am open to reasonable criticism about my work”).

Participants rated the frequency of their feedback behaviors on a 6-point Likert scale ranging from 1 (never) to 6 (always). Greater feedback literacy engagement is reflected by elevated scores on this measure, which has previously shown excellent psychometric validity within Chinese populations (Zhu et al., 2025). The overall internal reliability for our investigation remained strong, with a Cronbach's alpha reaching 0.873.

#### 2.2.2 Emotion and motivation self-regulation (T2)

Emotion and motivation self-regulation was assessed using the Chinese version of the emotion and motivation self-regulation questionnaire (EMSR-Q), which was originally developed by Alonso-Tapia et al. (2014) and revised for Chinese adolescents by Wang et al. (2025). The revised instrument consists of 19 items categorized into five dimensions: positive regulation of motivation (e.g., viewing learning tasks as challenges to be overcome), process-oriented self-regulation, performance-oriented self-regulation, avoidance-oriented self-regulation, and negative self-regulation of stress (e.g., “What instructions so long! They only make me confused”). These dimensions collectively reflect students' strategies for initiating, maintaining, and adjusting their emotional and motivational states during learning processes.

Participants rated the extent to which each item described their typical learning experience on a 5-point Likert scale ranging from 1 (completely inconsistent) to 5 (completely consistent). To derive a composite score representing adaptive self-regulation capabilities, items belonging to maladaptive dimensions (e.g., avoidance-oriented self-regulation and negative self-regulation of stress) were reverse-scored, such that higher aggregate scores indicate higher levels of effective emotion and motivation self-regulation. The Chinese version of the EMSR-Q has shown satisfactory validity and reliability among Chinese secondary school students (Wang et al., 2025). In the present study, the Cronbach's alpha coefficient for the scale was 0.837.

#### 2.2.3 Mathematics discourse feedback skills (T3)

Mathematics discourse feedback skills were assessed using the mathematics discourse feedback skills scale (MDFSS), which was specifically developed and validated for Chinese high school students by Chen et al. (2024). This 24-item instrument is designed to measure students' competency in engaging in discourse-based feedback processes and comprises six distinct dimensions: comparative analysis (four items; e.g., “I can analyze my weaknesses in math learning based on the feedback I receive”), Expressing Communication (four items; e.g., “I can use the language of mathematics and express the mathematical problems I encounter”), Mathematical Reasoning (three items; e.g., “I can use deductive reasoning to understand and apply feedback”), Monitor and Adjust (5 items; e.g., “I can adapt my math learning strategies based on feedback”), Diagnostic Evaluation (five items; e.g., “I can evaluate feedback to determine its usefulness in improving my math learning performance”), and Implementation Capacity (three items; e.g., “I can formulate feasible plans to translate feedback from math learning into actionable steps”).

Participants rated the extent to which they agreed with each statement on a 6-point Likert scale ranging from 1 (strongly disagree) to 6 (strongly agree). Higher aggregate scores reflect a higher level of proficiency in utilizing mathematical discourse to process and enact feedback. The MDFSS has demonstrated excellent construct validity and reliability within the Chinese educational context (Chen et al., 2024). In the present study, the Cronbach's alpha coefficient for the total scale was 0.875.

#### 2.2.4 Mathematical higher-order thinking (T1, T2, T3)

Mathematical higher-order thinking was assessed using the mathematical higher-order thinking scale (H-MHOTS), developed and validated specifically for Chinese high school students by Zhou et al. (2024). This 45-item instrument is designed to capture the multidimensional nature of higher-order thinking in mathematics and comprises four distinct dimensions: mathematical critical thinking (e.g., “I am good at approaching math problems in a systematic and organized way”), mathematical creative thinking (e.g., “I can solve different types of math problems in different ways”), mathematical problem-solving skills (e.g., “I can reason logically and rigorously based on definitions and laws”), and mathematical metacognitive skills (e.g., “I know if I have mastered the math content I have learned”).

Participants rated the extent to which each statement described their actual mathematical learning situations on a 5-point Likert scale ranging from 1 (not at all) to 5 (fully consistent). Higher scores indicate a higher level of mathematical higher-order thinking capabilities. The scale has demonstrated satisfactory structural validity and reliability within the Chinese senior high school context (Zhou et al., 2024). In the present study, the Cronbach's alpha coefficient for the total scale was 0.912.

### 2.3 Data analysis strategy

All statistical procedures were executed sequentially utilizing SPSS (IBM Corp., Armonk, NY, USA) and Mplus (Muthén & Muthén, Los Angeles, CA, USA). Initial examinations focused on handling non-responses and subject dropout. To determine the underlying mechanism of unobserved values across the three longitudinal waves, Little's missing completely at random test was applied (Little, 2024). Because the assumption of MAR was supported, we handled the missing data using the full information maximum likelihood (FIML) estimation method in Mplus. FIML is highly recommended for longitudinal structural equation modeling as it utilizes all available data points to produce unbiased parameter estimates without reducing statistical power (Baraldi and Enders, 2010). Furthermore, a sensitivity analysis was conducted comparing the FIML estimates with a completer-only dataset (using listwise deletion; Blozis et al., 2013). The comparison revealed no substantive differences in the direction, magnitude, or significance of the structural paths, thereby confirming the robustness of the FIML approach. Subsequently, potential attrition effects were scrutinized by contrasting the initial demographics and primary variables of retained participants against those who exited early. This comparison was achieved through Chi-square analyses and independent samples *t*-tests, successfully confirming the absence of systematic dropout bias. Second, prior to hypothesis testing, we evaluated the measurement models and addressed potential methodological artifacts. To assess common method bias (CMB), Harman's single-factor test was conducted using confirmatory factor analysis (CFA) by comparing the fit of the hypothesized multi-factor model against a single-factor model (Podsakoff et al., 2024). Additionally, to ensure that the measurement instruments operated equivalently across gender groups, multi-group confirmatory factor analysis (MGCFA) was executed (Immekus, 2021). Measurement invariance was tested sequentially across configural, metric, and scalar models (Cheung and Rensvold, 2002), evaluated primarily by changes in the comparative fit index (ΔCFI < 0.010) and root mean square error of approximation (ΔRMSEA < 0.015). Third, descriptive statistics and Pearson bivariate correlations were computed to examine the fundamental relationships among feedback literacy behavior at T1, emotion and motivation self-regulation at T2, mathematics discourse feedback skills at T3, and mathematical higher-order thinking across all three waves.

The ultimate analytical phase utilized structural equation modeling to evaluate the proposed single and sequential mediating mechanisms. In the SEM analysis, latent variables were employed rather than average scale scores. Given the large number of items in our measurement instruments (e.g., 45 items for the H-MHOTS), we utilized the item parceling technique to maintain an optimal parameter-to-sample size ratio, reduce random measurement error, and improve model convergence (Henseler, 2010). Specifically, dimension-level parceling was applied, where the mean scores of the established subscales served as the manifest indicators for their respective latent constructs. Furthermore, regarding path specification, the structural model was specified to test all possible forward-in-time direct and indirect paths among the predictor, mediators, and outcome variables. No paths were arbitrarily omitted; rather, the full structural model was estimated simultaneously. Alongside these estimated paths, we incorporated autoregressive parameters into our structural framework to stringently separate longitudinal directional pathways. This meant explicitly adjusting for preceding measurements of mathematical higher-order thinking. Following the recommendations of Hu and Bentler (Hu and Bentler, 1999), acceptable model fit was determined via the Chi-square value, Tucker–Lewis index (TLI), CFI, standardized root mean square residual (SRMR), and RMSEA. Furthermore, the evaluation of indirect trajectories relied on a bias-corrected bootstrapping technique generating 5,000 samples. According to Hayes (2013), these mediated effects achieved statistical significance when their corresponding 95% confidence intervals entirely excluded zero.

## 3 Results

### 3.1 Attrition analysis

Of the initial 1,795 participants at T1, 1,220 students (68.0%) completed all three waves of the survey. To examine whether the participant attrition (32.0%) was systematic, we conducted a series of attrition analyses comparing the “completers” (*n* = 1,220) and the “dropouts” (*n* = 575). Initial Chi-square analyses confirmed demographic equivalence between the retained cohort and those who exited early. Specifically, the two groups showed no significant discrepancies in their baseline distributions of gender, academic grade, residential setting, and parents' educational attainment (all *p*s >0.05). Second, independent samples *t*-tests were conducted to compare the baseline (T1) levels of the key study variables. The results indicated that dropouts did not differ significantly from completers in their initial levels of feedback literacy behavior, emotion and motivation self-regulation, mathematics discourse feedback skills, or mathematical higher-order thinking (all *p*s >0.05). Finally, Little's Missing Completely at Random (MCAR) test was performed to examine the overall pattern of missing data across the three time points. The test yielded a non-significant result (χ^2^ = 29.761, df = 27, *p* > 0.05), providing statistical evidence that the missing data mechanism was MCAR (Enders, 2022). Collectively, these findings suggest that attrition was random and unlikely to introduce systematic bias into the study's conclusions.

### 3.2 Measurement invariance

To ensure that the measurement instruments operated equivalently across gender groups, we conducted MGCFA (Vandenberg and Lance, 2000). Measurement invariance was tested sequentially for the four key constructs: feedback literacy behavior, emotion and motivation self-regulation, mathematics discourse feedback skills, and mathematical higher-order thinking. Following the recommended guidelines (Cheung and Rensvold, 2002), invariance was considered established if the change in CFI (ΔCFI) was less than 0.010 and the change in RMSEA (ΔRMSEA) was less than 0.015 between nested models.

As detailed in Table 1, multi-group analyses confirmed measurement equivalence across gender for every studied variable. Initially, the unconstrained baseline models exhibited adequate fit, establishing identical underlying factor patterns for both boys and girls (configural invariance). Subsequently, introducing equality constraints on the factor loadings yielded no substantial decrement in fit (ΔCFI ≤ 0.001; ΔRMSEA ≤ 0.010), thus supporting metric invariance. Furthermore, simultaneous restriction of both item intercepts and loadings produced scalar models that maintained acceptable fit parameters. These minimal deviations from the partially constrained models (ΔCFI ≤ 0.001; ΔRMSEA ≤ 0.005) firmly established strong equivalence.

**Table 1:** Goodness-of-fit indices for measurement invariance tests across gender.

Construct/Model	χ^2^	*df*	CFI	RMSEA	Δχ^2^	Δdf	ΔCFI	ΔRMSEA
Feedback literacy behavior (T1)								
Configural model	18.28	10	0.998	0.030	–	–	–	–
Metric model	20.31	14	0.999	0.022	2.05	4	0.001	−0.008
Scalar model	23.97	18	0.999	0.019	3.66	4	0	−0.003
Emotion and motivation self-regulation (T2)								
Configural model	21.94	10	0.997	0.041	–	–	–	–
Metric model	23.68	14	0.998	0.031	1.27	4	0.001	−0.01
Scalar model	26.46	18	0.998	0.026	2.81	4	0	−0.005
Mathematics discourse feedback skills (T3)								
Configural model	16.33	18	1	0	–	–	–	–
Metric model	20.23	23	1	0	3.90	5	0	0
Scalar model	22.32	28	1	0	2.11	5	0	0
Mathematical higher-order thinking (T3)								
Configural model	3.41	4	1	0	–	–	–	–
Metric model	6.91	7	1	0	3.50	3	0	0
Scalar model	10.47	10	1	0.009	3.59	3	0	0.009

CFI, comparative fit index; RMSEA, root mean square error of approximation. All Δχ^2^ tests were non-significant (*p* > 0.05).

Specifically, for the outcome variable, mathematical higher-order thinking, the scalar invariance model showed an excellent fit (χ^2^ = 10.47, df = 10, CFI = 1.000, RMSEA = 0.009), and the comparison with the metric model indicated strict invariance (Δχ^2^ = 3.59, *p* > 0.05; ΔCFI = 0.000). Similar patterns of invariance were observed for feedback literacy behavior, emotion and motivation self-regulation, and mathematics discourse feedback skills. These findings confirm that the constructs possess the same meaning and scaling properties across gender groups, thereby justifying meaningful mean comparisons and structural equation modeling in subsequent analyses.

### 3.3 Common method bias

Given that the data for all study variables were collected through self-report measures, CMB could potentially inflate the relationships between constructs. To mitigate this issue, we employed both procedural and statistical remedies. In terms of procedural remedies, we adopted a longitudinal design with a three-wave data collection strategy, creating a temporal separation between the predictor, mediator, and outcome variables. This temporal lag helps reduce the likelihood that respondents' transient moods or response styles at a single time point would systematically bias the associations among variables (Podsakoff et al., 2012). Additionally, we ensured the anonymity of the participants and emphasized that there were no right or wrong answers to reduce social desirability bias (Nederhof, 1985).

To address potential statistical biases, a confirmatory factor analysis was utilized to perform Harman's single-factor assessment. Following Malhotra et al. (2006), this procedure evaluates if one underlying dimension explains most of the shared variance across instruments. Specifically, the adequacy of our proposed four-factor framework was contrasted with an alternative structure that forced all observed items to load on just one factor. The results showed that the hypothesized four-factor model fitted the data excellently (χ^2^ = 398.72, df = 146, CFI = 0.985, TLI = 0.982, RMSEA = 0.035, SRMR = 0.024). In contrast, the single-factor model exhibited a very poor fit to the data (χ^2^ = 9568.62, df = 152, CFI = 0.435, TLI = 0.365, RMSEA = 0.186, SRMR = 0.138). The significant deterioration in model fit suggests that a single factor cannot explain the variance in the data (Jordan and Troth, 2020). Therefore, common method bias is unlikely to be a pervasive threat to the validity of the study's findings.

### 3.4 Descriptive statistics

Table 2 presents the means, standard deviations, and bivariate correlations for the study variables. The results indicated that all variables were significantly correlated in the hypothesized directions. Specifically, feedback literacy behavior at T1 was positively associated with the mediator at T2, emotion and motivation self-regulation (*r* = 0.415, *p* < 0.01), and the mediator at T3, mathematics discourse feedback skills (*r* = 0.411, *p* < 0.01). Furthermore, Feedback Literacy Behavior (T1) showed significant positive correlations with mathematical higher-order thinking at both T2 (*r* = 0.374, *p* < 0.01) and T3 (*r* = 0.401, *p* < 0.01). These results provide preliminary support for H1. Regarding the longitudinal stability of the outcome variable, mathematical higher-order thinking demonstrated significant autoregressive associations across the three waves (T1–T2: *r* = 0.535, *p* < 0.01; T2–T3: *r* = 0.494, *p* < 0.01). This confirms the importance of controlling for prior levels of higher-order thinking in the subsequent analysis to rigorously examine the effects of the predictors.

**Table 2:** Means, standard deviations, and correlations among study variables.

Variable	*M*	SD	1	2	3	4	5	6
1. Feedback literacy behavior (T1)	3.877	0.747	–					
2. Mathematical higher-order thinking (T1)	4.252	0.534	0.313^**^	–				
3. Emotion and motivation self-regulation (T2)	3.566	0.678	0.415^**^	0.116^**^	–			
4. Mathematical higher-order thinking (T2)	4.281	0.497	0.374^**^	0.535^**^	0.308^**^	–		
5. Mathematics discourse feedback skills (T3)	4.709	0.776	0.411^**^	0.126^**^	0.510^**^	0.215^**^	–	
6. Mathematical higher-order thinking (T3)	4.279	0.465	0.401^**^	0.279^**^	0.390^**^	0.494^**^	0.390^**^	–

^**^p < 0.01.

### 3.5 Mediating effects of emotion and motivation self-regulation

To test H2, which posited that emotion and motivation self-regulation mediates the relationship between feedback literacy behavior and mathematical higher-order thinking, we examined the structural paths within the longitudinal mediation model. The structural model demonstrated an excellent fit to the data: χ^2^ = 419.57, df = 340, *p* < 0.001, CFI = 0.996, TLI = 0.996, RMSEA = 0.011, and SRMR = 0.034.

The standardized path coefficients and indirect effects are presented in Table 3. After controlling for the autoregressive effects of the variables across time points, Feedback Literacy Behavior at T1 significantly and positively predicted Emotion and Motivation Self-Regulation at T2 (β = 0.471, *p* < 0.001). In turn, emotion and motivation self-regulation at T2 significantly predicted mathematical higher-order thinking at T3 (β = 0.118, *p* = 0.002).

**Table 3:** Standardized structural path coefficients and indirect effects.

Structural path	Bias-corrected bootstrapped estimates for the effects			
	β	SE	*p*	95% CI
Direct paths					
Feedback literacy behavior (T1) → emotion and motivation self-regulation (T2)	0.471	0.024	<0.001	[0.422, 0.517]
Emotion & motivation self-regulation (T2) → mathematical higher-order thinking (T3)	0.118	0.037	0.002	[0.044, 0.190]
Feedback literacy behavior (T1) → mathematical higher-order thinking (T3) (*c*′)	0.139	0.036	<0.001	[0.068, 0.210]
Indirect effect					
Feedback literacy (T1) → emotion and motivation self-regulation (T2) → higher-order thinking (T3)	0.056	0.018	0.002	[0.022, 0.090]

β, standardized coefficient; SE, standard error; CI, confidence interval; *c*′, direct effect.

To assess the significance of the mediation effect, we examined the specific indirect effect derived from the product of the structural paths. The analysis revealed a significant specific indirect effect of feedback literacy behavior on mathematical higher-order thinking via emotion and motivation self-regulation (β = 0.056, *p* = 0.002). Additionally, the direct effect of feedback literacy behavior (T1) on mathematical higher-order thinking (T3) remained significant (β = 0.139, *p* < 0.001), suggesting a partial mediation effect. These findings support H2, indicating that students with higher feedback literacy behavior are more likely to engage in adaptive emotion and motivation self-regulation, which subsequently fosters their mathematical higher-order thinking.

### 3.6 Serial mediating effects

**Figure 2:**
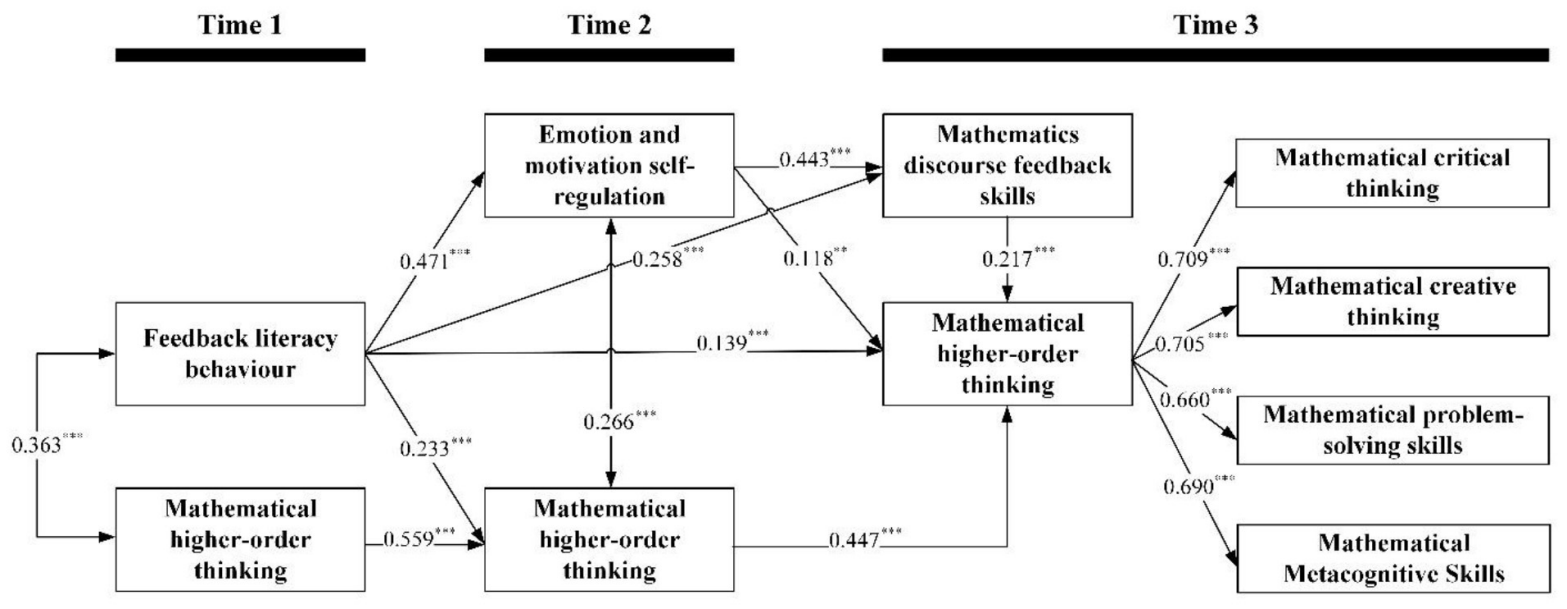
Standardized path estimates for the final serial mediation model. ^**^p < 0.01; ^***^p < 0.001.

To test the serial mediation hypothesis (H4), which proposed that feedback literacy behavior at T1 influences mathematical higher-order thinking at T3 through the sequential mediation of emotion and motivation self-regulation at T2 and mathematics discourse feedback skills at T3, we estimated the structural equation model while controlling for autoregressive effects. The standardized path coefficients and explained variances (*R*^2^) are presented in Figure 2 and Table 4.

**Table 4:** Standardized and unstandardized path estimates for the serial mediation model.

Variables	T2 emotion and motivation self-regulation		T3 mathematics discourse feedback skills		T2 mathematical higher-order thinking		T3 mathematical higher-order thinking	
	(*R*^2^ = 0.189)		(*R*^2^ = 0.362)		(*R*^2^ = 0.432)		(*R*^2^ = 0.479)	
	*B*	β (SE)	*B*	β (SE)	*B*	β (SE)	*B*	β (SE)
T1 feedback literacy behavior	0.405	0.435^***^ (0.025)	0.274	0.260^***^ (0.031)	0.148	0.233^***^ (0.028)	0.087	0.139^***^ (0.036)
T2 emotion and motivation self-regulation			0.499	0.441^***^ (0.029)			0.078	0.118^**^ (0.037)
T3 mathematics discourse feedback skills							0.127	0.217^***^ (0.037)
T1 mathematical higher-order thinking					0.499	0.559^***^ (0.027)		
T2 mathematical higher-order thinking							0.439	0.447^***^ (0.034)

B, unstandardized path coefficient; β, standardized path coefficient; SE, standard error; *R*^2^, squared multiple correlation (variance explained). The model controlled for autoregressive effects (i.e., prior levels of the outcome variables) as shown in the table.

^**^p < 0.01.

^***^p < 0.001.

The path analysis results revealed significant positive associations along the hypothesized serial chain. Specifically, feedback literacy behavior at T1 significantly and positively predicted emotion and motivation self-regulation at T2 (β = 0.435, *p* < 0.001). Subsequently, emotion and motivation self-regulation at T2 was a strong positive predictor of mathematics discourse feedback skills at T3 (β = 0.441, *p* < 0.001). Finally, mathematics discourse feedback skills at T3 significantly and positively predicted mathematical higher-order thinking at T3 (β = 0.217, *p* < 0.001). Furthermore, the model explained a substantial proportion of variance in the endogenous variables, accounting for 18.9% of the variance in emotion and motivation self-regulation, 36.2% in mathematics discourse feedback skills, and 47.9% in mathematical higher-order thinking at T3.

To evaluate the statistical significance of the mediated pathways, a bias-corrected bootstrap procedure involving 5,000 iterations was applied (see Table 5). The results confirmed a significant specific serial indirect effect of feedback literacy behavior on mathematical higher-order thinking via emotion and motivation self-regulation and mathematics discourse feedback skills (Ind. = 0.045, *SE* = 0.009, 95% CI [0.030, 0.064]). As the 95% confidence interval did not contain zero, the serial mediation pathway was statistically significant.

**Table 5:** Bias-corrected bootstrapped indirect effects of the serial mediation model.

Indirect effects	Bias-corrected bootstrapped estimates for the effects			
	*B*	β	SE	95% CI
T1 feedback literacy behavior → T2 Emotion and motivation self-regulation → T3 mathematical higher-order thinking	0.035	0.056	0.018	[0.022, 0.090]
T1 feedback literacy behavior → T2 mathematical higher-order thinking → T3 mathematical higher-order thinking	0.065	0.104	0.015	[0.077, 0.136]
T1 feedback literacy behavior → T3 mathematics discourse feedback skills → T3 mathematical higher-order thinking	0.035	0.056	0.012	[0.035, 0.081]
T1 Feedback literacy behavior → T2 emotion and motivation self-regulation → T3 mathematics discourse feedback skills → T3 mathematical higher-order thinking	0.028	0.045	0.009	[0.030, 0.064]

B, unstandardized indirect effect; β, standardized indirect effect; SE, standard error; CI, confidence interval. Results are based on 5,000 bootstrap samples. The 95% CI is reported for the standardized indirect effects.

Additionally, other indirect pathways were also significant. The specific indirect effect via emotion and motivation self-regulation alone was significant (Ind. = 0.056, 95% CI [0.022, 0.090]). Similarly, the path via mathematics discourse feedback skills alone was also significant (Ind. = 0.056, 95% CI [0.035, 0.081]), which supports H3. Notably, the direct effect of feedback literacy behavior at T1 on mathematical higher-order thinking at T3 remained significant (β = 0.139, *p* < 0.001) after accounting for all mediators.

In conclusion, these findings support H4, indicating that feedback literacy behavior not only directly promotes mathematical higher-order thinking but also exerts its influence indirectly through a sequential process: it first enhances students' ability to regulate their emotions and motivation, which in turn fosters their engagement in constructive mathematics discourse, which is concurrently linked to improved higher-order thinking. The significant direct effect suggests that this serial mediation is partial.

## 4 Discussion

### 4.1 Summary of findings

This research primarily aimed to explore how feedback literacy practices longitudinally influence advanced mathematical cognition within a sample of Chinese adolescents. By tracking data across three distinct time points, we tested a sequential mediating framework that incorporated the regulation of emotions and motivation, alongside mathematics discourse feedback capabilities. Following adjustments for baseline demographics and prior autoregressive levels, our findings yielded support for the hypothesized theoretical pathways.

First, consistent with H1, our findings revealed a significant positive association between feedback literacy behavior at T1 and mathematical higher-order thinking at subsequent time points. This suggests that students' proactive engagement with feedback serves as a critical predictor for the long-term development of complex cognitive capabilities in mathematics.

Second, the structural equation modeling results supported the mediating roles proposed in H2 and H3. Specifically, emotion and motivation self-regulation (T2) was found to mediate the relationship between feedback literacy behavior (T1) and mathematical higher-order thinking (T3). This indicates that feedback literacy behavior fosters a regulated psychological state, which in turn facilitates cognitive growth. Similarly, mathematics discourse feedback skills (T3) acted as a significant mediator, suggesting that the behavioral habit of processing feedback translates into cognitive gains through the enhancement of communicative and argumentative competencies in mathematics.

Most importantly, supporting H4, the study confirmed a serial mediation pathway: feedback literacy behavior (T1) → emotion and motivation self-regulation (T2) → mathematics discourse feedback skills (T3) → mathematical higher-order thinking (T3). This complete causal chain highlights a progressive developmental trajectory where feedback literacy behavior first helps students establish emotional and motivational stability, which subsequently empowers them to engage in constructive mathematical discourse, a skill set that is concurrently associated with higher-order thinking skills. Additionally, the direct effect of feedback literacy behavior on mathematical higher-order thinking remained significant, indicating that the identified mediators partially explain the underlying mechanism.

### 4.2 Theoretical implications

The present study offers several significant theoretical contributions to the literature on feedback literacy, self-regulation, and mathematics education by elucidating the longitudinal mechanisms underlying cognitive development.

First, this study extends self-regulated learning theory by empirically validating feedback literacy behavior as a robust longitudinal predictor of mathematical higher-order thinking. While previous research has largely conceptualized feedback literacy as a facilitator of immediate task performance or academic achievement, our findings suggest that it serves a more profound function: acting as a developmental catalyst for cognitive restructuring. By establishing a temporal link between early feedback engagement (T1) and subsequent higher-order thinking (T2, T3), this study provides evidence that feedback literacy behavior is not merely a reactive strategy for error correction but a proactive self-regulatory resource that accumulates over time to reshape students' cognitive schemas. This aligns with and expands upon the view that active engagement with feedback is a prerequisite for deep learning, moving the theoretical focus from the “uptake of information” to the “development of thinking capabilities” (Nieminen and Carless, 2023).

Second, the confirmation of the emotional mediation pathway enriches control-value theory within the context of high-stakes assessment. Our findings demonstrate that feedback literacy behavior functions as a critical antecedent that enhances students' emotion and motivation self-regulation. Theoretically, this implies that the behavioral act of seeking and processing feedback provides students with a sense of agency, which alters their cognitive appraisals from helplessness to control (Ye et al., 2025). This study bridges the gap between behavioral engagement and affective regulation, suggesting that feedback literacy operates as a protective mechanism. It mitigates the resource-depleting effects of negative emotions (e.g., anxiety regarding failure) and preserves the cognitive resources necessary for higher-order thinking, thereby supporting the resource allocation model in explaining the “affective cost” of complex mathematical learning (Barroso et al., 2021; Guo et al., 2025c).

Third, the study advances the sociocultural perspective on mathematics learning by identifying mathematics discourse feedback skills as a pivotal mechanism connecting internal behavior to cognitive outcomes. Supporting Vygotsky's notion that higher mental functions originate on the social plane, our results highlight that feedback literacy behavior must be translated into “socially shared cognition” through discourse to fully impact higher-order thinking. This finding theoretically distinguishes between the “intrapersonal” processing of feedback (silent reflection) and the “interpersonal” negotiation of meaning, or articulating reasoning (Nicol, 2021). It suggests that the theoretical construct of feedback literacy should be broadened to explicitly include discursive competencies, as the ability to verbalize and debate mathematical logic is what ultimately internalizes feedback into advanced reasoning skills (Boud and Dawson, 2023).

Furthermore, the present study provides a nuanced perspective on the theoretical intersection between the individual-level nature of SRL and the socially constructed nature of the Sociocultural Perspective. While integrating these frameworks is a strength of our model, it also reveals inherent theoretical tensions. On one hand, a potential conflict exists: SRL often emphasizes individual psychological safety and the internal protection of self-efficacy, which can sometimes manifest as withdrawal from social interactions to avoid exposing incompetence. In contrast, sociocultural processes inherently demand public vulnerability, where students must expose their mathematical errors and misconceptions for collaborative discourse. On the other hand, our serial mediation model illustrates a synergistic resolution to this conflict. By establishing that emotion and motivation self-regulation precedes mathematics discourse feedback skills, our findings suggest that successful sociocultural co-construction is fundamentally contingent upon prior individual-level regulation. In essence, effective intra-psychological regulation serves as the necessary affective buffer that allows students to overcome the inherent interpersonal risks of social participation. This progressive transition reconciles the tension between individual self-protection and social learning demands, demonstrating how internal regulatory stability paves the way for external dialogic engagement.

Finally, the verification of the serial mediation model integrates the aforementioned theories into a cohesive “Affect–behavior–cognition” framework. By linking emotion and motivation self-regulation to mathematics discourse feedback skills, this study provides longitudinal support for broaden-and-build theory in an educational setting (Carmona-Halty et al., 2021). The serial pathway illustrates a theoretical hierarchy: regulated emotions broaden the students' momentary thought-action repertoires, enabling them to engage in the socially risky behavior of public discourse, which subsequently builds enduring cognitive resources, specifically higher-order thinking (Zhao et al., 2024). This integrated model offers a nuanced explanation of how learning occurs over time, proposing that emotional stability is the foundational bedrock that empowers social interaction, which in turn fuels cognitive advancement (Darling-Hammond et al., 2020). This sequential perspective challenges isolated theoretical views and advocates for a holistic understanding of student development where affective, social, and cognitive processes are inextricably intertwined (Osher et al., 2020).

### 4.3 Practical implications

The empirical results of this research offer practical guidance for teachers, instructional developers, and educational authorities seeking to cultivate advanced mathematical reasoning among secondary school students.

First, given the foundational role of feedback literacy behavior at T1 in predicting long-term cognitive development, educators are encouraged to prioritize the explicit instruction of feedback literacy as a core pedagogical goal rather than treating it as a peripheral skill. Teachers are advised to shift their focus from merely providing detailed comments to equipping students with the skills to use them. Practically, this can be achieved by integrating feedback protocols into regular mathematics instruction. For instance, after an exam or assignment, teachers could require students to complete an error analysis log where they are guided to classify their errors, articulate the gap between their current performance and the standard, and formulate a specific revision plan (Stanton et al., 2021). This compels students to move beyond passive score-checking to active engagement strategies, such as seeking, making sense of, and using information, thereby initiating the cognitive scaffolding process identified in this study (Nieminen and Carless, 2023).

Second, the mediating role of emotion and motivation self-regulation at T2 underscores the necessity of creating emotionally supportive assessment environments. In the context of high-stakes testing in China, where students often experience high anxiety and fear of losing face due to academic failure, teachers can leverage feedback as a tool for emotional scaffolding (Liu and Xin, 2025). Instructional feedback should be framed to support mastery goals, which focus on learning for understanding, rather than performance goals that focus solely on scores (Wisniewski et al., 2020). Teachers should encourage students to view feedback not as a judgment of ability but as a mechanism for gaining control over their learning (Nieminen et al., 2022). By guiding students to proactively seek help and manage their affect, educators can help students down-regulate anxiety and maintain the motivational resilience required for complex problem-solving (Camacho-Morles et al., 2021).

Third, the significance of mathematics discourse feedback skills at T3 as a proximal predictor of higher-order thinking suggests a need for a paradigm shift from solitary correction to dialogic feedback in the classroom. The results indicate that internalizing feedback requires externalization through language. Therefore, mathematics classrooms should incorporate structured opportunities for students to verbalize their mathematical reasoning (Sedova et al., 2019). Teachers can implement peer feedback circles or think-aloud protocols where students are required to explain their correction strategies to peers or debate alternative solution paths (Papadopoulos et al., 2022). This social practice allows students to negotiate meaning and detect logical inconsistencies, effectively transforming the behavioral habit of feedback uptake into deep cognitive processing (Nicol, 2021).

Finally, the validation of the serial mediation model implies that interventions should be holistic and developmentally sequenced. A piecemeal approach focusing solely on emotional regulation or solely on drilling difficult problems may be insufficient. Effective curriculum design could benefit from considering the developmental trajectory revealed in this study: first, establishing robust habits of feedback usage to build confidence and agency; second, striving to support students' emotional and motivational regulation; and third, encouraging rich mathematical discourse. By aligning instructional practices with this causal chain from feedback literacy behavior to emotion and motivation self-regulation, then to mathematics discourse feedback skills, and finally to mathematical higher-order thinking, educators can more effectively cultivate the higher-order thinking skills essential for students' future academic success.

Importantly, recognizing the inherent variability of human actors and educational systems, these instructional recommendations are not prescriptive panaceas. Educators are encouraged to adapt these strategies to fit their specific cultural, demographic, and contextual classroom environments to optimize student outcomes (Forgas and O'Driscoll, 1984).

### 4.4 Limitations and future directions

Despite the significant theoretical and practical implications, several limitations of the present study should be acknowledged to guide future research. First, the sample was recruited via convenience sampling from three senior high schools in Hebei Province, China. This sampling approach may limit the generalizability of the findings to broader populations, such as students in other geographical regions, distinct cultural contexts, or different educational tiers like junior high schools and higher education institutions (Emerson, 2015). Future research should aim to employ stratified random sampling across diverse cultural and socio-economic backgrounds to cross-validate the proposed serial mediation model (Le et al., 2023).

Second, all key variables in this study were assessed using self-report questionnaires. Although rigorous statistical procedures confirmed that common method bias was unlikely to be a pervasive threat, self-reported data may still be susceptible to memory recall errors or social desirability bias, particularly when adolescents evaluate their own cognitive and behavioral capacities (Brutus et al., 2013). To enhance the robustness of these findings, future studies would benefit from incorporating multi-informant assessments, such as teacher evaluations of students' mathematics discourse feedback skills, or objective performance metrics, including standardized assessments for mathematical higher-order thinking, to triangulate the results (Mudarra et al., 2022).

Third, while the three-wave longitudinal design with 3-month intervals successfully established temporal precedence for the primary pathways, it may not capture the complete dynamic trajectory of cognitive and affective development. Specifically, both the second mediator, mathematics discourse feedback skills, and the final outcome variable, mathematical higher-order thinking, were measured concurrently at T3. This concurrent measurement restricts the ability to draw strict causal inferences for the final link in the serial model. Consequently, although our data adequately fit the hypothesized serial mediation pathway, alternative or competing configurations (e.g., reverse causality or parallel processes) cannot be entirely ruled out. Future research could adopt panel designs with more measurement waves spanning a longer duration, such as multiple academic years, or utilize intensive longitudinal methods like ecological momentary assessment to capture micro-level fluctuations in emotion and motivation self-regulation (Laurenceau et al., 2025).

Finally, the current theoretical framework primarily focused on intra-individual psychological and behavioral mechanisms, potentially overlooking critical contextual boundary conditions. Factors at the interpersonal or environmental level, such as teacher feedback literacy, classroom goal structures, and baseline mathematical achievement, were not explicitly controlled beyond autoregressive effects (Lehikoinen et al., 2025). Future studies should consider utilizing multilevel modeling to investigate how classroom-level or teacher-level variables might moderate the pathways from feedback literacy behavior to complex cognitive outcomes (Liu et al., 2025).

## 5 Conclusion

In conclusion, the present three-wave longitudinal study elucidates the complex developmental mechanisms linking feedback literacy behavior to mathematical higher-order thinking among Chinese high school students. The findings confirm that proactive engagement with feedback serves as a direct catalyst for advanced cognitive development. Furthermore, this relationship is sequentially mediated by intra-individual psychological regulation and interpersonal social interaction. Specifically, early feedback literacy behavior fosters adaptive emotion and motivation self-regulation, which subsequently empowers students to engage in constructive mathematics discourse. This progressive trajectory is ultimately linked to the deep cognitive processing required for mathematical higher-order thinking. Theoretically, this study integrates self-regulated learning, control-value theory, and sociocultural perspectives into a cohesive framework, highlighting the intertwined nature of affective, behavioral, and cognitive processes. Practically, the results emphasize the necessity for educators to transcend traditional corrective feedback by actively cultivating students' feedback literacy, providing emotional scaffolding, and facilitating dialogic feedback environments. By prioritizing these interconnected elements, educational practices can more effectively nurture the sophisticated mathematical reasoning essential for students' long-term academic success.

## Data Availability

The raw data supporting the conclusions of this article will be made available by the authors, without undue reservation.
